# How Can Access to Mental Health Services in Switzerland Be Improved in the Aftermath of the COVID-19 Pandemic?

**DOI:** 10.3389/phrs.2025.1607659

**Published:** 2025-07-02

**Authors:** Camilla Sculco, Marco Meneguzzo, Emiliano Albanese

**Affiliations:** Institute of Public Health, Università della Svizzera Italiana, Lugano, Switzerland

**Keywords:** mental health, common mental disorders, health services use, Switzerland, COVID-19

## Abstract

**Background:**

The global spread of the COVID-19 pandemic posed exceptional challenges for society and healthcare systems, with adverse effects on population’s mental health. Understanding the pandemic’s impact on mental health and service use in Switzerland is a priority, along with outlining evidence-based recommendations to improve access and support for those in need.

**Analysis:**

Studies from Switzerland report a growing burden of psychological distress, especially among vulnerable groups such as children, adolescents, young women, socially isolated individuals, and those with pre-existing health conditions. The pandemic also caused variations in mental health service use across the country.

**Policy Options:**

[1] Strengthening mental health services for vulnerable populations. [2] Re-structuring mental health services and their capacity to cope with the increasing demand. [3] Enhancing prevention and promotion of mental health and wellbeing.

**Conclusion:**

In the aftermath of the pandemic, there is a need to strengthen and restructure mental health services, enhance prevention and promotion efforts, and integrate mental health into pandemic preparedness to mitigate the long-term impacts of future public health emergencies.

## Background

The World Health Organization (WHO) defines mental health (MH) as a state of wellbeing, in which a person can realize his or her abilities, cope with stressors of life, be productive and contribute to his or her community [1]. The concept is integral to that of health; hence, mental wellbeing is essential for an individual’s overall health. The global spread of the COVID-19 pandemic posed exceptional challenges for societies and healthcare (HC) systems, with adverse effects on population’s MH. These effects were exerted both directly - by the disease infection and its consequences on the body - as well as indirectly by the restrictions imposed as public health (PH) measures [2]. In fact, published research suggests that the SARS-CoV-2 leads to cognitive, emotional, neurovegetative, and behavioural harms [3]; this may happen because the virus enters the brain directly or triggers an immune response [4]. Environmental stressors, such as loss of control, fear of infection, and isolation, substantial changes in daily life [5] medical and financial uncertainties, all contributed to the rise in MH problems in the population, including stress and anxiety [6, 7].

Switzerland is known for having a developed HC system that emphasizes access to high-quality medical services, including psychiatric and psychological care [8]. The system is based on mandatory health insurance; both medical and psychological treatments are often covered by insurance [9], although specific coverage varies regionally. Indeed, switzerland lacks a unified national MH policy or care planning: responsibilities are divided among the federal government, cantons, and municipalities. MH services are provided by both public and private organisations (i.e., hospitals, outpatient clinics, practitioners in private practice, home treatment, etc.) that vary in structures and configurations across cantons and regions. The local health service planning is organised by cantonal governments that also run public facilities; private providers and non-profit providers offer various levels of care, from outpatient counselling to inpatient treatment. While compulsory health insurance regulates payments for psychiatric services nationwide, there are variations in funding structures: inpatient care is co-financed by health insurers and cantons through taxes, whilst outpatient care is fully covered by health insurers. Intermediate services (like day clinics, home treatment, or case management) lack a legal framework and consistent funding at the federal level, leading to significant regional disparities [10]. Switzerland prioritizes community-based and outpatient services rather than inpatient care, which promote early intervention and the provision of socio-psychiatric support within the local community. Further, the system promotes a multidisciplinary approach, involving psychiatrists, psychologists, social workers, and nurses [8].

Compared to other European countries, Switzerland experienced a liberal implementation of mitigation measures to halt SARS-CoV-2 transmission [11, 12]. No strict lockdown with stay-at-home orders was implemented [11] and the mitigation strategy was largely based on individual responsibility. Freedom of movement was maintained, and shops, businesses, and schools remained open. Sanitary measures introduced early on were also lifted relatively quickly in the spring and summer of 2020 [12]. At the end of 2020, Switzerland’s score on the Oxford COVID-19 Government Response Tracker Stringency Index was 37.5 - substantially lower than those of its neighbouring countries. Germany, Italy, and France reported significantly higher scores of 60.7, 66.7, and 78.7 respectively, indicating the implementation of more stringent mitigation measures [12]. The Country’s handling of the pandemic was deemed relatively effective both from a PH and from an economic perspective [13]. Still, a growing body of evidence highlights the longer-term consequences of the pandemic on the population’s MH, which are of increasing concern for the HC systems [14].

All things considered, it is crucial to fully understand how the pandemic impacted population MH and the use of MH services in Switzerland and to outline evidence-based recommendations to improve access to MH services for those in need.

## Analysis

### Growing Burden and Increased Prevalence of Mental Health Problems in the Aftermath of the COVID-19 Pandemic

Evidence supports the association between the COVID-19 pandemic and MH problems [13, 14] and studies conducted in Switzerland witness a growing burden and increasing prevalence of psychological distress including depression, anxiety, post-traumatic stress, and disturbed sleep [15] among specific population groups such as the youth and women [16]. One survey conducted during the pandemic on a sample of a thousand individuals within the three linguistic regions, revealed that one-third (34%) of respondents had worsened mental wellbeing, including depressive mood, anhedonia, feelings of anxiety and loneliness [17] compared to the period before the pandemic. Similarly, in a population-based longitudinal study carried out in the canton of Zurich to measure the prevalence of MH problems at least 6 months after SARS-CoV-2 infection, more than half of participants reported symptoms of fatigue and one in four had symptoms of depression [18]. Similar effects were described in the general population [19], as well as in vulnerable individuals [2, 20, 21] but the burden was not homogeneous across population groups [22].

### Heterogeneous Burden Across Population Groups

In Switzerland, the pandemic has exacerbated pre-existing social, economic and health inequalities across population groups [7, 22, 23]. Indeed, vulnerable groups including individuals living alone or lacking support from family and friends, individuals with pre-existing mental or physical illness, low socio-economic status, or low educational attainment experienced increased psychological distress [2, 5, 17, 18, 20] and lower level of wellbeing compared to the rest of the population [10]. Further, children, adolescents and young women appeared significantly more affected by the negative consequences of the pandemic [16]. Research conducted during the pandemic revealed that a quarter of Swiss youths reported symptoms of Common Mental Disorders (CMDs) (i.e., depression: 17.7%; anxiety: 13.2%) [24] and young women reported stress symptoms more often compared to older and male individuals [18]. The researchers also found that low educational level and unemployment were positively associated with symptoms of depression [18]. Interestingly, one study revealed that in Switzerland, during the pandemic, the type of residential environment (urban vs. rural) influenced population’s mental wellbeing [25]; here, living in urban areas was associated with increased probability of higher psychological distress compared to living in rural environments. Indeed, the environmental and social conditions of urban areas may challenge MH in different ways: on the one hand, urban areas offer opportunities for socializing, education, culture, work, and easy access to care; on the other hand, urban living includes limited presence of greenspace and nature, easier access to drugs, exposure to crime and violence, poverty, pollution, traffic, loneliness, and increased need for handling distress [13, 25].

### Variation in the Use of Mental Health Services Following the COVID-19 Pandemic

Data regarding the use of psychiatric and psychotherapeutic services collected during the first pandemic years (2020 and 2021) revealed a decline in outpatient consultations yet an increase in hospitalization rates among children and adolescents. Similarly, there was evidence of an increase in outpatient and inpatient treatments and hospitalizations for suicide attempts in these years [26, 27]. One study carried out using nationwide hospital and insurance claim data, revealed the impact of the pandemic on the use of MH services: the study observed an increase in mental HC use, in admissions for depressive and neurotic disorders and in antidepressant and anxiolytic drug claims in females aged <20 years. The study also revealed an increase in antipsychotic medication prescriptions in both females <20 years and those aged 20–30 years [7]. Similarly, a survey conducted on a representative sample of the general population presented raising demand for psychological support and helpline calls, especially from younger citizens [17]. Interestingly, a study carried out at the University Hospital of Geneva (HUG) aimed at investigating differences in admissions at a Swiss psychiatric Emergency Department (ED) between the pandemic and pre-pandemic period; here, researchers documented a reduction in the total number of psychiatric admissions to ED during the pandemic and this trend seemed to be associated with living alone and presence of severe pre-existing psychopathologies [15]. Overall, changes in services provision varied significantly across regions [28]: in certain areas, MH services delivery was preserved with a comprehensive range of services and minor adjustments due to hygienic and physical distancing measures [28]. Other regions experienced disruption as services were re-organised due to shutdowns and closures of facilities such as rehabilitation programs, ambulatory clinics, and outreach services [28].

## Policy Options

### Strengthening Mental Health Services for Vulnerable Populations

Research emphasizes the burden that COVID-19 has placed on the MH of vulnerable populations in Switzerland [1, 5, 15] (Table 1); a high level of economic support and a high-quality HC system in view of moderate containment measures as applied in Switzerland did not sufficiently protect the entire population to the same extent from the adverse psychological impact of the pandemic [13]. Further, ignoring the adverse effects of a prolonged pandemic on mental wellbeing could result in negative consequences for the life trajectories (e.g., education and career, intra-national migration, family and health) of vulnerable groups [13]. This underscores the need for alerting MH professionals, authorities, and health policymakers to take measures to address the gaps in access and use of MH services for vulnerable population, while continuing efforts in the prevention of CMDs and suicide [26, 27]. Likewise, it is urgent to improve MH monitoring systems by ensuring the collection of high-quality, comprehensive data, particularly in areas where gaps remain, such as children epidemiology. Strengthening data collection not only provides a clearer picture of the current MH status of the population but also enables evidence-based decision-making. Aligned with this, it is essential to establish effective structures and processes to turn data into practical actions. This includes creating mechanisms for the agile development, testing, and implementation of innovative care models. By closing the gap between research and practice, Switzerland can respond more effectively to emerging needs and deliver timely, targeted interventions that improve MH outcomes across population groups.

**TABLE 1:** Recommendation 1. to improve access to mental health services in Switzerland in the aftermath of the COVID-19 pandemic (Canton Ticino, Switzerland. 2024).

Recommendation	Core elements
Strengthening mental health services for vulnerable populations	• Person-centred approaches to care
	• Community-centred approaches to care

#### Person-Centred Approaches to Care

MH services should be person-centred: person-centeredness is an approach that prioritizes the individual needs, preferences, and values of the person receiving care. It emphasizes a collaborative and respectful relationship between HC professionals and patients, acknowledging the autonomy of patients and involving them in decision-making processes. Person-centred care is seen as a shift away from a more traditional, provider-focused model towards a model that places the individual at the centre of the care process. This approach is associated with improved patient satisfaction, better outcomes, and increased adherence to treatment plans [29].

#### Community-Centred Approaches to Care

MH services should have a community-centred orientation: such a system would involve community psychiatry with a territorial and proximity-oriented focus. Measures based on peer support, leveraging existing friendships and acquaintances are recommended; these may include the establishment of patient support groups and networks for vulnerable individuals such as peer support from people who have successfully outlived mental illness and help others to recover [30]. Similarly, they can take inspiration from the micro-community model that involves voluntarily maintaining close social contacts with a small, fixed group of individuals, without changing members, as proposed by sociological studies conducted during the pandemic [28]. As mentioned, urban-rural behavioural differences and socio-economic factors play a role in determining differences in wellbeing, and coping behaviours of individuals living in Switzerland [5]. Likewise, individuals with CMDs may have smaller social networks than the general population, which is a disadvantage in terms of social support and inclusion [28]. Hence, community-centred approaches to care should support users to enlarge and maintain their social networks.

### Re-Structuring Mental Health Services and Their Capacity to Cope With the Increasing Demand

#### Avoid Institutionalised Settings and Provide Treatment and Care to Patients in a Flexible Way, Especially in Outpatient and Outreach Settings

Evidence indicates the need for re-organising and re-structuring MH services, specifically focusing on promoting early intervention and short-term hospitalizations [15] (Table 2). Re-structuring of MH services is advisable also in view of a shortage of care professionals and a growing demand. Similarly, evidence emphasizes the importance of providing care by avoiding institutionalised settings: in fact, as inpatient care may be unnecessary, costly, and harmful [31], pandemic-adapted MH services necessitate a strong outpatient component [28] that is organised on a smaller spatial scale [8]. Within this context, several countries, such as the United Kingdom, proposed an alternative service model to reduce the pressure on ED and psychiatric wards: referred to as Mental Health Decision Units (MHDUs) (or short-stay crisis unit) and are designed to reduce the strain on EDs and inpatient admissions. MHDUs operate 24/7 and have a maximum stay of 12–72 h, they are staffed by experienced MH nurses and HC assistants, with oversight from psychiatrists; their staff-to-patient ratio is high, with approximately one staff member every two patients [31].

**TABLE 2:** Recommendation 2. to improve access to mental health services in Switzerland in the aftermath of the COVID-19 pandemic (Canton Ticino, Switzerland. 2024).

Recommendation	Core elements
Re-structuring mental health services and their capacity to cope with the increasing demand	• Avoid institutionalised settings and provide treatment and care to patients in a flexible way, especially in outpatient and outreach settings
	• Integrated socio-psychiatric services
	• Whole-of-Society approach to mental health

#### Integrated Socio-Psychiatric Services

Evidence supports the use of integrated socio-psychiatric interventions [18] organized within local communities encouraging collaboration across roles and breaking down barriers between care professionals [31]. Here, the strengthening of territorial services to minimise travelling time for seeking care is implied. One example is the extension of home treatment projects e.g., supported houses services established in the cantons of Zurich and Bern [32, 33] to treat patients commonly referring to inpatient settings at home. Another approach, also known as primary care behaviourist model, integrates a MH professional into primary care [30] to increase screening, surveillance, and diagnosis of CMDs [25]. Further, interventions ought to integrate community health workers that deliver non-clinical MH interventions such as psycho-education and stress management training to primary care. Additionally, long-term strategies, including the introduction and maintenance of helplines are advised.

#### Whole-Of-Society Approach to Mental Health

The concept “Whole-of-Society” (WoS) [34] defines the collective effort of different actors involved in the policy making to achieve shared goals by overcoming siloed works. This approach can be put into practice through stakeholder dialogue or focus group discussions. The approach aims to address the challenges posed by the Social Determinants of Health (SDH) and by fostering collaboration, governance, and action across sectors, while engaging non-state actors in collaboration with governments. Indeed, the approach acknowledges the influence of the SDH on (mental) health and it recognizes that (mental) health is highly dependent on sectors beyond HC. Relevant stakeholders to be involved in collaborations include patient associations, families, communities, intergovernmental organisations, religious institutions, civil society, academia, the media, voluntary associations, and the private sector. The approach encourages stakeholders involved in the policy making, including both governments and non-state actors to develop integrated policies and programs by increasing policy coherence and effectiveness; further, it emphasizes stakeholders’ accountability for MH impact [35].

### Enhancing Prevention and Promotion of Mental Health and Wellbeing

Good MH contributes greatly to the wellbeing of individuals, families, communities, and society (Table 3). Promoting MH is essential, as it strengthens the resources needed to enhance wellbeing. This goes beyond a purely clinical approach and requires a broader strategy that includes social determinants (e.g., income, ethnicity, education level, gender) to reduce inequalities that affect health. Therefore, MH promotion should be implemented at the individual, social, and societal levels. Structural measures to improve the social determinants of health, such as reducing poverty and loneliness, and providing additional resources to children of parents with mental disorders are also crucial for addressing the issues.

**TABLE 3:** Recommendation 3 to improve access to mental health services in Switzerland in the aftermath of the COVID-19 pandemic (Canton Ticino, Switzerland. 2024).

Recommendation	Core elements
Enhancing prevention and promotion of mental health and wellbeing	• Investing on education and public health campaigns to reduce stigma surrounding mental health
	• Targeting prevention and promotion strategies to vulnerable populations

#### Investing in Education and Public Health Campaigns to Reduce Stigma Surrounding Mental Health

To increase awareness on MH issues and perceived needs, it is recommended to promote MH literacy, particularly regarding clinical symptoms and opportunities for seeking help [24]; this involves promoting open dialogue on MH in schools and workplaces [24]. Measures to be implemented include those directed at early detection and suicide prevention as well as facilitating access to low-threshold counselling and information services [12, 16]. One example is the Swiss Mental Health First Aid program [36] at the workplace: it is a program of the Swiss Foundation Pro Mente Sana that since 2019 has been offering first aid courses for counsellors in Switzerland. The program instructs individuals on providing first aid to friends, family members, or colleagues facing CMDs symptoms until professional help is available; similarly, first aiders are trained to address the existing stigma towards individuals suffering from MH problems [36]. School-based programs that focus on developing social and emotional skills have been proven to positively influence children’s and adolescents' behaviour and academic performance when properly implemented. Studies also shows the effectiveness of school programs in reducing symptoms of CMDs. It is therefore suggested to integrate MH promotion into school curricula and to raise awareness among school staff accordingly [37]. In the same way, it is suggested to promote healthy practices for social media use in schools. Identifying and encouraging best practices has become increasingly important to prevent problematic behaviours related to social media. Experts recommend the following measures: 1. Promoting media literacy from an early age, ideally starting in primary school; this includes teaching children and adolescents how to recognize reliable sources, as well as equipping them with strategies to navigate the overwhelming flow of information on social media. 2. Creating safe spaces, within schools or youth programs, where young people, especially girls, can discuss social media content and its impact on MH, particularly on issues like body image [37].

#### Targeting Prevention and Promotion Strategies to Vulnerable Populations

To mitigate health inequalities across cultural and socio-economic groups, preventive interventions and promotion strategies should be tailored according to vulnerable population’s needs and lifestyles [13]. Hence, it is relevant to learn more about the underlining causes of MH problems in the population. Well-established theories show that living conditions and social factors such as poverty, racism, and discrimination significantly impact psychological wellbeing. Individuals from marginalized social groups or those who face major challenges during childhood and adolescence are at greater risk of developing MH disorders later in life. Peer support programs can be introduced to train children and adolescents in providing mutual support, alongside coaching and mentorship initiatives that offer guidance [38]. School-wide and workplace-wide MH screenings and interventions are required to address the low recognition of needs and service use among children, adolescents, and young adults [30], helping them to recognise warning signs, moderate to severe symptoms of CMDs and overcoming barriers to access services [24]. Indeed, MH education is essential for positive changes in health, social behaviour, and coping strategies [13]. One example is the so-called Early Childhood Mental Health Consultation Intervention which involves MH providers partnering with schoolteachers for regular check-ins [30]. To widen coverage, MH and wellbeing ought to be promoted through various outreach campaigns and media channels, including radio, TV, and social media, ensuring accessibility for diverse population segments [17]. Given that young people spend a significant amount of time online, digital and media-based interventions are particularly impactful [39]. These include MH apps that promote mindfulness [40], journaling, and mood tracking; social media campaigns aimed at normalizing conversations around MH [41]; and online therapy platforms and helplines, which are rapidly gaining popularity [42].

## Conclusion

The COVID-19 pandemic significantly impacted MH and the use of related services in Switzerland, highlighting the urgent need to improve prevention, promotion and access to care for vulnerable populations. Following a detailed analysis, we developed evidence-informed recommendations for policymakers aimed at strengthening and re-structuring MH services, as well as enhancing preventive and promotion efforts. The COVID-19 also highlighted the need for pandemic preparedness that includes a strong focus on MH; in Switzerland, future preparedness plans must integrate MH prevention as a core component to ensure timely support during crises. This involves re-organising MH services, building community resilience, and developing rapid response strategies to address psychological problems, and social isolation. Proactive planning can help mitigate the long-term MH impacts of future PH emergencies, especially among vulnerable groups such as children, adolescents, and the elderly.
